# Regular Doses of Nature: The Efficacy of Green Exercise Interventions for Mental Wellbeing

**DOI:** 10.3390/ijerph17051526

**Published:** 2020-02-27

**Authors:** Mike Rogerson, Carly Wood, Jules Pretty, Patrick Schoenmakers, Dan Bloomfield, Jo Barton

**Affiliations:** 1 School of Sport, Rehabilitation and Exercise Sciences, University of Essex, Wivenhoe Park, Colchester, Essex CO4 3SQ, UK; ppscho@essex.ac.uk (P.S.); jobarton@essex.ac.uk (J.B.); 2 School of Life Sciences, University of Westminster, 115 New Cavendish Street, London W1W 6UW, UK; C.Wood@westminster.ac.uk; 3 School of Life Sciences, University of Essex, Wivenhoe Park, Colchester, Essex CO4 3SQ, UK; jpretty@essex.ac.uk; 4 College of Life and Environmental Sciences, University of Exeter, Penryn Campus, Treliever Road, Penryn, Cornwall TR10 9FE, UK; D.Bloomfield@exeter.ac.uk

**Keywords:** green exercise, mental wellbeing, interventions, health, environment, interventions

## Abstract

This study investigated the efficacy of medium-term Green Exercise (GE; being physically active within a natural environment) interventions for improving wellbeing, by pooling data collected at the start and end of participants’ engagement with a range of GE interventions. Hypotheses were that (i) interventions would show good efficacy for improving wellbeing in the overall sample; (ii) compared to participants reporting ‘average to high’ wellbeing at the start of their project, participants with ‘low’ starting wellbeing would report greater improvements post-intervention; and (iii) improvements would significantly differ between age groups. The pooled dataset was categorized in line with UK norms (*n* = 318) and analyzed using a standardized meta-analysis approach. Effect size was large: g = 0.812 (95% CI [0.599, 1.025]), and differences in wellbeing changes associated with project duration, age or sex were not statistically significant. Compared to those reporting ‘average-high’ starting wellbeing, participants reporting ‘low’ starting wellbeing exhibited greater improvements (BCa 95% CI [−31.8, −26.5]), with 60.8% moving into the ‘average-high’ wellbeing category. GE can play an important role in facilitating wellbeing and can provide alternative pathways for health and social care practice. Public health commissioners should consider integrating such interventions for patients experiencing low wellbeing or associated comorbidities.

## 1. Introduction

The last decade has seen a growing number of studies evidencing the health and wellbeing benefits of ‘green exercise’ (GE; being physically active within a natural environment or greenspace), which provides greater physical and mental health benefits than physical activity (PA) or nature contact alone [1,2,3,4,5,6,7,8]. GE reduces stress, depression and blood pressure, increases self-esteem, mood and wellbeing, and enhances heart rate variability. These benefits seem to be universally obtainable, with evidence of health and wellbeing improvements in children and adolescents [9], adults [1,2,3,4,5,6,7,8] and vulnerable cohorts including disaffected youth [10], adults living with dementia [11] and those experiencing physical and/or mental ill-health such as post-traumatic stress disorders [12]. Wellbeing is important to health because it increases life expectancy, improves recovery from illness [13,14] and is associated with positive health behaviours. The UK has lower wellbeing compared to some of its European counterparts, incurring additional costs to the UK economy [15]. Thus, GE interventions might provide a complementary pathway for facilitating wellbeing improvements [15]. This process may involve two intertwining pathways [16]; (i) simple exposure to natural environments is salutogenic, and (ii) natural environments facilitate pro-wellbeing behaviours, such as PA, through the behavioural invitations (opportunities) or ‘affordances’ of their characteristics [17,18]. 

There is a need to better understand who can benefit most from regular ‘doses’ of GE, and what the optimal doses might be for maximising wellbeing in different cohorts. This evidence can inform public health priorities, urban and rural planning and recommendations for healthy lifestyles. A meta-analysis evidenced that acute bouts of nature exposure were associated with increased positive effect [19]. Furthermore, a multi-study analysis evidenced that participation in a single bout of GE was associated with greater improvements in self-esteem (d = 0.68) and mood (d = 0.56) for participants with self-declared mental ill-health compared to those who were already healthy (self-esteem d = 0.41, mood d = 0.53) [2]. Similarly, individuals with mental ill-health experienced significantly greater reductions in stress following a rural walk than people with good mental health [20]. 

Whereas most GE research has focused on single bouts of GE, cross-sectional research has reported links between access to greenspaces, PA and self-reported health and wellbeing. Access to nature can function equigenically, helping to balance health and wellbeing across socioeconomic differences within populations [21]. Relationships between neighbourhood greenspace, health and wellbeing are sometimes partially mediated by PA levels, although research differences pervade the area, and there is no consensus about when this occurs, and how and when other factors interact [22,23,24,25].

Situated between these two paradigms, academic research has not yet addressed the efficacy of medium-term (12 weeks to 1 year) GE behaviours or interventions for improving mental wellbeing. In practice, GE interventions are often small scale and data are seldom collected across multiple time-points, leading to small data samples that are often not large enough to yield meaningful statistical analyses. Pooling datasets from multiple small-scale projects enables more worthwhile analyses whilst statistically accounting for sample sizes. The current study aimed to analyse the efficacy of medium-term GE interventions for improving wellbeing, by pooling data collected at the start and end of participants’ engagement with a range of GE interventions.

Informed by the findings of Barton and Pretty [2], we hypothesised that (i) GE interventions would show good efficacy for improving wellbeing in the overall participant sample (demonstrated by moderate to large effect sizes); (ii) compared to participants reporting ‘average to high’ wellbeing at the start of their project, participants with ‘low’ starting wellbeing would report greater improvements post-intervention; and (iii) improvements would significantly differ between age groups.

## 2. Materials and Methods

### 2.1. Participants and Projects

Using purposive sampling, participants were adult attendees to one of six wellbeing projects in the UK that focused on interaction with nature, between January 2010 and September 2017 (*n* = 318; 125 males, 177 females, 16 did not report their sex; aged 43.17 ± 15.34 years, 37 participants did not report their age). All participants provided informed consent for their providing of data for research purposes; and where required, ethical approval was granted by the University of Exeter ethics committee prior to the commencement of each of the projects (dates of approval: 18/5/2010; 3/5/2012; 22/8/2012; 18/11/2014; 13/1/2015). Whereas some projects were attended by both the general public and individuals with defined needs, other projects were attended only by individuals with defined needs. All projects combined nature-based activities with other therapeutic approaches such as counselling and were free to attend.

Project A (*n* = 13; 10 males, 3 females; aged 37.5 ± 11.8 years) comprised a variety of conservation, ecotherapy, and craft-focused interventions across the UK, that were attended by the general public and individuals with defined needs, such as learning disabilities, mental ill-health, recovery from hospital stays, low confidence, anxiety, psychiatric disorders, depression and risk behaviours. Project A ran between February 2016 and February 2017 and was 12 weeks in duration.

Project B (*n* = 20; 7 males; 13 females; aged 39.9 ± 7.9 years) aimed to improve the physical and mental wellbeing of vulnerable adults through community gardening and food growing activities within a UK city. It comprised multiple interventions across the city that were attended by the general public and individuals with defined needs such as autism, learning disabilities, mental ill-health, physical ill-health or impairments, homelessness, alcohol or substance misuse. Project B ran between January 2015 and September 2017 and was 12 weeks in duration.

Project C (*n* = 7; 3 males; 4 females; aged 20.9 ± 1.8 years) was a wilderness therapy-based project that sought to engage and positively change the lives of young people who typically face multiple barriers to success. These barriers included low self-esteem and mental wellbeing, poverty, abusive or ineffective families, drug and alcohol abuse, school failure, and youth offending orders. Many participants had previously experienced significant social, psychological or physical trauma, and some entered the programme at a stage where they were self-harming. Project C focused particularly on participants’ self-esteem and self-worth, because of their strong influences upon many facets of their lives. The project blended wilderness therapy with other counselling techniques, centered around weekend and weeklong wilderness expeditions. Project C ran between April 2012 and December 2016 and was 26 weeks induration.

Project D (*n* = 12; 8 males; 3 females; 1 did not say; aged 38.5 ± 11.2 years) was a community-based project for adults experiencing mental health difficulties that aimed to enable participants to increase their aspirations, personal responsibility and ability to undertake challenges in their lives. The project combined elements of wilderness therapy and walking programmes, in the form of weekly, facilitated walks whereby participants walked together in a group, exploring and learning about countryside and coastal environments and wildlife. Project D also incorporated opportunities to camp in scenic and remote natural environments around the UK. Project D ran between January and December 2010 and was 26 weeks induration.

Project E (*n* = 208; 75 males; 118 females; 15 did not say; aged 44.6 ± 16.0 years) was a city-based community-wide project that comprised a variety of community activities such as community food growing, helping vulnerable groups to access nature, reducing the carbon footprint, tree planting, developing community and therapeutic gardens and helping the homeless. Its aims included supporting communities experiencing low wellbeing and PA levels and reducing inequalities in wellbeing across the city. Project E ran between January and November 2015 and was 26 weeks in duration.

Project F (*n* = 58; 22 males; 36 females; aged 45.0 ± 15.1 years) comprised eight different health and wellbeing interventions across two cities and other locations in the southwest UK. Interventions were partnerships between health staff working in primary care, local organisations owning and/or managing natural assets, and practitioners; and involved groups of four to ten participants. Each weekly session included walking and conservation or tasks with silent or meditative elements, and were based in woodland areas, coastal zones, countryside dominated by agriculture and greenspace in and around urban settlements. Project E ran between March 2015 and October 2016 and was 12 weeks induration.

### 2.2. Design, Measures and Data Processing

Participants reported their wellbeing via questionnaires completed at the start (first week) and end (final week) of the project. Wellbeing was measured using the Warwick–Edinburgh Mental Well-Being Scale (WEMWBS), which comprises a global wellbeing measure including affective-emotional aspects, cognitive-evaluative dimensions and psychological functioning. In Projects A, C, D and F, wellbeing data were collected using the full version of the WEMWBS, which consists of 14 positively worded items that address positive aspects of mental health [26]. It is scored by summing responses to each item, which are scored on a five-point Likert scale from 1 (none of the time) to 5 (all of the time). Overall scores range from 14 to 70, with higher scores indicating better wellbeing. The scale is validated for use in both adults and adolescents in the UK. The original scale validation study reported a Cronbach’s alpha of 0.91 for a UK sample [27], and more recently a value of 0.92 has been reported for England population-level data [28]. WEMWBS scores correlate with indexes of happiness and general health, and low scores can be predictive of depression [28]. In Projects B and E, wellbeing data were reported via the Short Warwick–Edinburgh Mental Well-Being Scale (SWEMWBS), which consists of seven items from the full scale. Overall scores range from 7 to 35, with higher scores indicating better wellbeing. A Cronbach’s alpha of 0.84 has been reported for SWEMWBS using recent England population-level data [28], and correlation between the WEMWBS and the SWEMWBS has been calculated to be 0.954 [29]. Raw SWEMWBS scores were converted to metric scores in accordance with Stewart-Brown et al. [29] prior to further data processing and analyses.

### 2.3. Categorising Wellbeing Scores

In order to contextualise the reported raw WEMWBS and metric-converted SWEMWBS scores, they were categorised in relation to UK population mean and standard deviation (SD) values. Scores within one SD of the mean were considered as ‘average’, scores more than one SD below the mean were categorised as ‘low’, and scores more than one SD above the mean were categorised as ‘high’ [28].

Wellbeing scores were categorised in line with the Health Survey England (HSE) 2016 data (published Dec 2017) relating to the respective versions of the scale. As mean and SD SWEMWBS values were not published in HSE’s report, these were calculated using the full published dataset, including only data whereby participants completed all items with a 1–5 score (i.e., no missing values or answering ‘don’t know’). For WEMWBS, the categories were calculated as ‘Low’ 14–38; ‘Average’ 39–61; ‘High’ 61–70. For SWEMWBS, the categories were calculated as ‘Low’ 7.00–18.59; ‘Average’ 19.25–26.02; ‘High’ 27.03–35.00. For analyses, categorised (low; average; high) wellbeing scores were then dichotomised to create categories of ‘low wellbeing’ and ‘average to high wellbeing’. 

Data for age were categorised in line with Barton and Pretty [2], creating four age categories (≤30 years, 31–50, 51–70, and ≥70 years of age).

### 2.4. Creating a Single Variable for Analyses

In order to create and use a single, amalgamated variable for the analysis of changes in wellbeing across the projects, the data from the respective scales were ‘normalised’ to ‘percentage scores’ that represented scores as percentage of the scale on which they were reported, using the following formula:

Percentage Score = ((wellbeing score − minimum score for respective scale)/range of respective scale) × 100

Short form: Percentage Score = ((wellbeing score − 7)/28) × 100

Full form: Percentage Score = ((wellbeing score − 14)/56) × 100

### 2.5. Statistical Analysis

Whole sample. Standardised meta-analysis methodology was used to assess changes in reported wellbeing (Δ wellbeing) across the whole sample from start to end of engagement. Mean pre and post-intervention wellbeing scores were entered into Comprehensive Meta-Analysis Version 3 (Biostat, Englewood, NJ, USA), by intervention, for multi-study analysis. Data were pooled to calculate an overall intervention effect estimate. This represents the weighted average of the combined individual intervention effects. To reduce the imprecision of the pooled-effect estimate, the inverse-variance method was used to assign weights to each project, so that larger projects with smaller standard errors were given more weight than smaller projects with larger standard errors. As the various project interventions took different approaches, the combined intervention effect estimates were calculated using a random-effects model meta-analysis [30,31]. 95% confidence intervals were calculated on the basis of the standard error of the pooled intervention effect. Statistical significance was set at *p* < 0.05. In addition to the overall meta-analysis, random-effects model moderator analyses examined the influence of project duration on the Δ wellbeing.

Following multi-study analysis, in line with Barton and Pretty [2], further analyses examined the effects of individual differences on the intervention-associated changes in wellbeing scores. The factors identified and available for this analysis were: sex (male, female); age group (in line with Barton and Pretty: ≤30 years, 31–50, 51–70, and ≥70 years of age); starting wellbeing status (low, average- high). Due to non-normality of data, non-parametric tests were used to examine the effects of these factors on the Δ wellbeing. A Kruskal–Wallis test was used to examine the effect of age group, and a Mann–Whitney U test was used to examine the effect of sex. As the distributions of data were significantly different between the dichotomised levels of wellbeing status, a bootstrapped (10,000 samples) independent samples t-test was used to examine effect of starting wellbeing status.

Low-wellbeing subsample. To investigate the efficacy of GE interventions in ‘low wellbeing’ adults, a multi-study analysis and follow-up analyses were conducted on data from participants who reported ‘low’ wellbeing pre-intervention. Due to a SD value of zero within the multi-study-analysis calculations, full Hedges’ g calculations were not possible when analysing pre- and post-intervention mean values. Therefore, the multi-study analysis examined the event rate of movement from reporting ‘low’ starting wellbeing status, to reporting ‘average-high’ end of intervention wellbeing status.

Similar to analyses of the whole sample, due to non-normality of data, non-parametric statistical tests were used to examine the effects of sex and age group on Δ wellbeing. A Kruskal–Wallis test was used to test the effect of age group, and a Mann–Whitney U test was used to examine the effect of sex.

## 3. Results

### 3.1. Whole Sample

**Figure 1:**
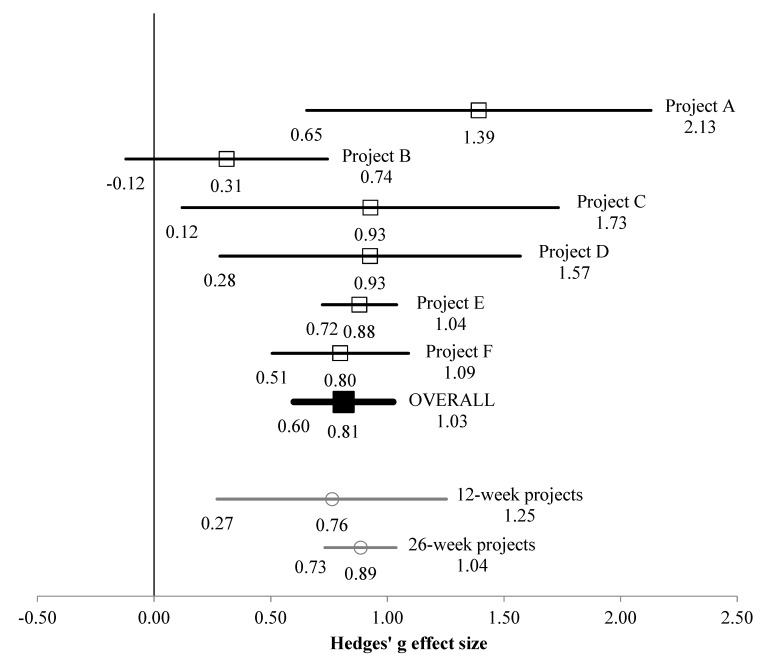
Multi-study effect sizes (±95% upper and lower limits) for start to end of intervention changes across the whole sample.

Mean and SD values by time-point across projects and samples are shown in Table 1. For the whole sample, the multi-study-analysis effect size was g = 0.812 (95% CI [0.599, 1.025], *p* < 0.001; see Figure 1), which can be considered to be large [32,33,34]. This is likely to be an overestimate [35], so should be interpreted with caution. Tests of heterogeneity found that variation in effect size between projects was not statistically significant (*p* = 0.138); the calculated I^2^ value indicated that 40% of the observed variance in Δ wellbeing scores between projects was due to real differences, with the remaining 60% due to sampling error, which represents low to moderate heterogeneity [36]. *A priori* moderator analysis found that there was not a statistically significant influence of project duration on Δ wellbeing. Further moderator analysis indicated that the wellbeing scale version used to measure wellbeing did not significantly affect the results within the multi-study analysis; see respective *Q* and *p* values in Table 2.

**Figure 2:**
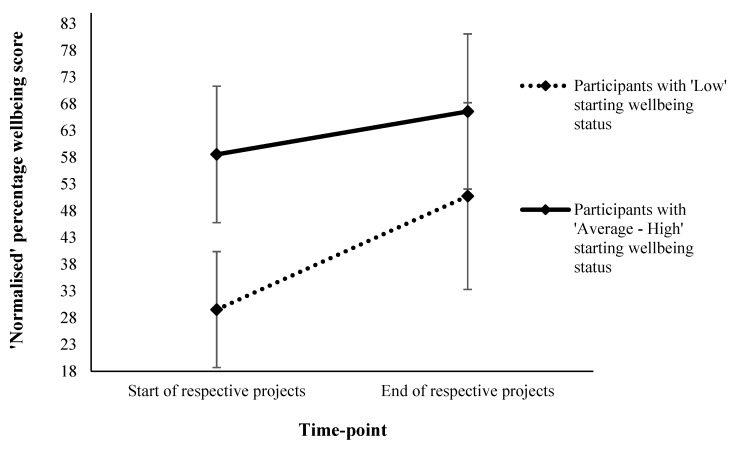
‘Normalised’ percentage wellbeing scores by time-point across projects and categorised starting wellbeing status.

A Mann–Whitney *U* test found that sex-associated differences in Δ wellbeing were not statistically significant (*U* = 10,601.0, Z = 0.618, *p* = 0.537), and a Kruskal–Wallis test found that age category-associated differences in Δ wellbeing were not statistically significant (X^2^_3_ = 3.296, *p* = 0.348; see Table 1). However, a bootstrapped (10,000 samples) independent sample t-test found that compared to those reporting ‘average to high’ starting wellbeing (in relation to national norms), participants reporting ‘low’ starting wellbeing exhibited a greater improvement in wellbeing post-intervention (t_316_ = 21.139, *p* = 0.001, BCa 95% CI [−31.8, −26.5]; see Table 3 and Figure 2).

### 3.2. Low Wellbeing Subsample

**Figure 3:**
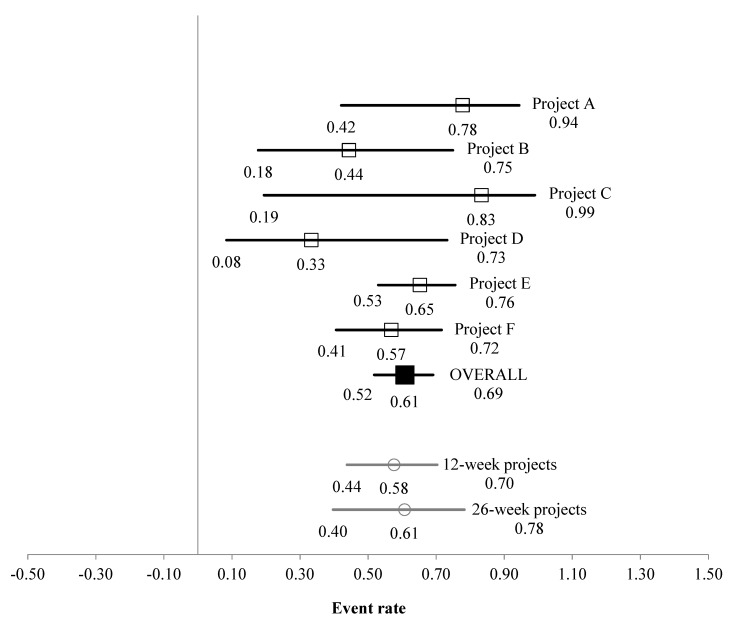
Multi-study event rates (±95% upper and lower limits) for ‘low’ starting wellbeing subsample moving to ‘average to high’ wellbeing post-intervention.

In total, 129 participants were categorised as having ‘low’ starting wellbeing (49 males, 71 females, 9 did not report their sex; aged 21.2 ± 18.7 years, 23 participants did not report their age). For the low wellbeing subsample, the multi-study-analysis event rate was 0.608 (95% CI [0.518, 0.691]), indicating that the interventions led to 60.8% of ‘low’ starting wellbeing participants moving into the average–high wellbeing group by the end of their project engagement (Figure 3). Tests of heterogeneity found that variation in effect size between projects was not statistically significant (*p* = 0.409); the calculated I^2^ value indicated that only 1.2% of the observed variance in Δ wellbeing scores between projects was due to real differences, with the remaining 98.8% due to sampling error, which represents low heterogeneity [36]. Other multi-study analysis statistics are presented in Table 2.

As with the whole sample, moderator analyses found that there were not statistically significant effects for project duration and wellbeing scale version used (see respective *Q* and *p* values in Table 2). A Mann–Whitney U test found that sex-associated differences in Δ wellbeing were not statistically significant (U = 1607.5, Z = −0.705, *p* = 0.481), and a Kruskal–Wallis test found that age category-associated differences in Δ wellbeing were not statistically significant (X^2^_3_ = 0.560, *p* = 0.905; see Table 1).

## 4. Discussion

This study investigated the efficacy of a range of GE interventions for improving wellbeing. Analysis showed the GE interventions to have good efficacy for improving wellbeing, thereby supporting hypothesis i. Across the pooled sample the interventions demonstrated a large effect size on a validated measure of wellbeing, although there are recent suggestions that such a high effect size in psychological research is likely to be a considerable overestimate [35], so should be interpreted with caution. More meaningfully then, the interventions produced a 61% event rate of ‘low’ starting wellbeing participants moving into the average–high wellbeing group. This finding is consistent with the notion that GE behaviours can facilitate wellbeing improvements in adults [1,2,3,4,5,6,7,8,16]. In terms of impacts of regular doses of participation, the current findings are also consistent with those concerning frequent participation in horticultural activity [37,38], perhaps unsurprisingly, as sometimes horticulture is considered as a ‘GE’ activity; although it is often considered distinct and therefore researched in its own right. Horticultural activities share key features with the projects included within the current study: social interaction, physical activity and interaction with nature. Soga et al.’s meta-analysis reported an effect size of 0.42 (95% CI [0.36, 0.48]). The difference in effect size reported in the current study and by Soga et al. may be a function of Soga et al.: (a) including multiple health and wellbeing outcome measures, whereas the current study focused on one; (b) including studies from seven countries (therefore being influenced by cross-cultural differences) whereas the current study was entirely UK-based; (c) including projects attended by individuals who were experiencing more extreme health, wellbeing-related and learning issues (compared to the current study), such as dementia and learning disabilities; and (d) including only horticulture activities whereas the current study included a range of GE activities. 

In support of hypothesis ii, individuals with lower starting wellbeing reported greater improvements at the end of the interventions than participants who reported ‘average to high’ starting wellbeing. This finding is consistent with Barton and Pretty’s findings on the influence of single bouts of GE on measures of self-esteem and mood [2]; and is also concurrent with the notion that nature can function equigenically across populations [21].

Hypothesis iii, that improvements would significantly differ between age groups, was not supported. However, the oldest age category reported the greatest wellbeing improvement, and this was consistent with the findings of McMahon et al.’s [19] meta-analysis, which examined effects of exposure to nature but did not address the extent of PA involved in experimental protocols. A key difference between the current study and previous work is that whereas the current study focuses on medium-term interventions, the studies of McMahon et al. [19] and Barton and Pretty [2] focused on acute bouts of GE. Further research examining single bouts and regular GE behaviours can elucidate possible reasons for variation in wellbeing and other related outcomes between individuals and single versus repeated bouts.

The lack of impact of project duration suggests that after 12 weeks, additional GE intervention weeks may maintain, but do not have additional benefit for wellbeing. The finding of low heterogeneity in effect size between projects both for the whole sample and the low starting wellbeing subsample indicates that GE interventions can be used efficaciously to achieve wellbeing improvements for range of different cohorts. Indeed, an implication of these findings is that GE interventions should be considered as a therapeutic approach for wellbeing for both the general population and vulnerable cohorts. As was implemented in Project F, health services can utilise GE as a non-pharmaceutical approach to complement existing treatments for the benefit of patients.

A strength of the current study is its use of data from real-world GE interventions. It reflects the extent of variation in the design of GE interventions and the outcomes of such practices. The current study offers a glimpse into the efficacy of GE interventions for wellbeing promotion and provides a methodological approach for similar future research. However, it is limited in the sample size and the number of interventions it comprised. Small sample sizes within studies also mean that estimated effect sizes are inclined to be less stable. As it becomes available additional data should be pooled in order to further elucidate the efficacies of different therapeutic GE approaches for wellbeing improvements. The current study engendered a quasi-experimental design without a control group. Randomised controlled trials and similar designs towards this level of rigour should be utilised to further examine the issue of GE interventions for wellbeing.

Like with research that has focused on single bouts of GE, wellbeing was measured at the start and end of the interventions. Research into mindfulness training and arts and crafts indicates that engaging with regular doses of these activity types can lead to positive neurological changes such as cortical volume as soon as 4 weeks into engagement, and that these changes correlate with self-reported improvements in feelings and wellbeing [39,40]. As participation in the doses of nature activities of the current study occurred over 12 weeks and participants reported improvements in wellbeing, it is plausible to suggest that positive neurological changes may have occurred. This possibility should be a focus of future research. Further, as wellbeing is a complex construct comprised of both eudaimonic and hedonic components, future research should address whether GE interventions are most effective at improving a specific wellbeing facet. Further to this point, whereas the current findings attest to the efficacy of the interventions for improving reported wellbeing, it is important to note that numerical measures do not reveal by which mechanisms the GE interventions improved wellbeing, or the key features of these interventions. There is evidence that social aspects of GE and created senses of purpose and achievement are key features towards wellbeing improvements [41,42,43,44]. Mixed-methods approaches could be used to rigorously map the lived experience, and the features and mechanisms of GE onto quantitative outcome findings. Better understanding of when, how and why GE participation works best for different cohorts can better inform the design of bespoke interventions, urban planning, and healthcare agendas. 

## 5. Conclusions

Whereas previous research evidences the importance of single bouts of GE for health and wellbeing outcomes, this multi-study analysis shows that medium-term GE interventions can play an important role in facilitating wellbeing. This novel approach of pooling data from multiple real-world GE interventions, and the unique analysis in relation to national data categories shows that individuals experiencing low wellbeing experience even greater improvements, thus supporting the use of these types of nature-based interventions as alternative pathways for health and social care practice. Public health commissioners should consider integrating such interventions for patients experiencing low wellbeing or associated comorbidities. The current findings suggest that such interventions may be particularly efficacious in older adults.

All procedures performed in studies involving human participants were in accordance with the ethical standards of the institutional and/or national research committee and with the 1964 Helsinki declaration and its later amendments or comparable ethical standards.

## Figures and Tables

**Table 1:** Raw, converted and ‘normalised’ mean ± SD values by project for whole sample and low wellbeing subsample.

	Scale	Project	Sample Size	Mean Raw/Metric-Converted Scores for Respective Scale *		Normalised Percentage Scores	
				Start of Intervention	End of Intervention	Start of Intervention	End of Intervention
Whole sample	WEMWBS (14–70)	A	13	33.3 ± 12.2	46.2 ± 9.0	34.5 ± 21.7	57.4 ± 16.2
		C	7	39.6 ± 12.8	57.9 ± 10.1	45.7 ± 22.9	78.3 ± 18.1
		D	12	36.0 ± 5.7	43.4 ± 6.2	39.3 ± 10.2	52.5 ± 11.1
		F	58	35.5 ± 13.1	45.6 ± 10.6	38.3 ± 23.4	56.4 ± 18.8
	SWEMWBS (7–35)	B	20	19.4 ± 2.7	20.5 ± 3.5	44.3 ± 9.6	48.3 ± 12.6
		E	208	21.2 ± 4.7	24.5 ± 4.8	50.6 ± 16.6	62.4 ± 17.0
Low wellbeing subsample	WEMWBS (14–70)	A	9	27.0 ± 6.0	43.9 ± 6.6	23.2 ± 10.6	53.4 ± 11.8
		C	2	23.0 ± 1.4	61.5 ± 12.0	16.1 ± 2.5	84.8 ± 21.5
		D	6	30.8 ± 2.1	40.7 ± 7.2	30.1 ± 3.8	47.6 ± 12.9
		F	37	27.2 ± 7.1	42.2 ± 10.3	23.6 ± 12.7	50.3 ± 18.3
	SWEMWBS (7–35)	B	9	16.9 ± 1.3	19.1 ± 4.3	35.4 ± 4.5	43.3 ± 15.3
		E	66	16.3 ± 2.4	21.3 ± 4.9	33.3 ± 8.6	50.9 ± 17.4

* SWEMWBS scores have been metric converted. ‘Low wellbeing subsample’ comprises participants who reported raw WEMWBS or metric-converted SWEMWBS scores categorised as ‘Low’ in relation to 2016 UK population data (WEMWBS: <39; SWEMWBS: <19.25).

**Table 2:** Multi-study analyses statistical values for the whole sample

		Test of Null (2-Tailed)		Heterogeneity between Projects/for Respective Moderator				Tau^2^		
		Z	*P*	*Q*	df (*Q*)	*P*	I^2^	Tau^2^	SE	Variance
**Whole sample**	Overall (in relation to Hedges g effect size)	7.463	<0.001	8.343	5	0.138	40.07	0.025	0.043	0.002
	Moderator effect for project duration	-	-	0.215	1	0.643	-	-	-	-
	12 weeks	3.053	0.002	-	-	-	-	-	-	-
	26 weeks	11.393	<0.001	-	-	-	-	-	-	-
	Including moderator of wellbeing scale version	-	-	1.086	1	0.297	-	-	-	-
	WEMWBS	7.287	<0.001	-	-	-	-	-	-	-
	SWEMWBS	2.240	0.025	-	-	-	-	-	-	-
**Low wellbeing subsample**	Overall (in relation to Event Rate)	2.345	0.019	5.060	5	0.409	1.19	0.003	0.169	0.029
	Including moderator of project duration	-	-	0.061	1	0.805	-	-	-	-
	12 weeks	1.081	0.280	-	-	-	-	-	-	-
	26 weeks	1.000	0.317	-	-	-	-	-	-	-
	Including moderator of wellbeing scale version	-	-	0.017	1	0.895		-	-	-
	WEMWBS	1.036	0.300	-	-	-	-	-	-	-
	SWEMWBS	1.188	0.235	-	-	-	-	-	-	-

**Table 3:** Mean ± SD starting and Δ normalised percentage values of respective scales by age category, sex and starting wellbeing status

		Sex		Age Category (years)				Starting Wellbeing Status	
		Male	Female	18–30	31–50	51–70	>70	Low	Average–High
**Whole sample**	**Starting**	47.2 ± 18.4	46.8 ± 17.5	48.6 ± 20.5	45.3 ± 16.2	49.8 ± 19.5	46.9 ± 12.4	29.5 ± 10.8	58.6 ± 12.8
	**Δ**	15.0 ± 16.5 *n* = 125	13.4 ± 15.8 *n* = 177	13.9 ± 16.9 *n* = 67	14.6 ± 17.1 *n* = 122	12.2 ± 15.3 *n* = 77	19.0 ± 14.1 *n* = 15	21.2 ± 18.7 *n* = 129	8.0 ± 12.1 *n* = 189
**Low wellbeing**	**Starting**	29.7 ± 11.0	30.7 ± 10.3	27.9 ± 11.0	30.4 ± 11.2	28.4 ± 11.0	36.5 ± 6.0	-	-
	**Δ**	22.6 ± 18.9 *n* = 49	21.3 ± 19.4 *n* = 71	23.9 ± 20.0 *n* = 25	21.9 ± 21.8 *n*= 49	21.9 ± 17.7 *n* = 25	23.2 ± 12.0 *n* = 7	-	-
